# Systematic identification and characterization of long non-coding RNAs in mouse mature sperm

**DOI:** 10.1371/journal.pone.0173402

**Published:** 2017-03-14

**Authors:** Xiaoning Zhang, Fengxin Gao, Jianbo Fu, Peng Zhang, Yuqing Wang, Xuhui Zeng

**Affiliations:** Institute of Life Science and School of Life Science, Nanchang University, Nanchang, China; University Hospital of Münster, GERMANY

## Abstract

Increasing studies have shown that mature spermatozoa contain many transcripts including mRNAs and miRNAs. However, the expression profile of long non-coding RNAs (lncRNAs) in mammalian sperm has not been systematically investigated. Here, we used highly purified RNA to investigate lncRNA expression profiles in mouse mature sperm by stranded-specific RNA-seq. We identified 20,907 known and 4,088 novel lncRNAs transcripts, and the existence of intact lncRNAs was confirmed by RT-PCR and fluorescence in situ hybridization on two representative lncRNAs. Compared to round spermatids, 1,794 upregulated and 165 downregulated lncRNAs and 4,435 upregulated and 3,920 downregulated mRNAs were identified in sperm. Based on the “Cis and Trans” RNA-RNA interaction principle, we found 14,259 targeted coding genes of differently expressed lncRNAs. In terms of Gene ontology (GO) analysis, differentially expressed lncRNAs targeted genes mainly related to nucleic acid metabolic, protein modification, chromatin and histone modification, heterocycle compound metabolic, sperm function, spermatogenesis and other processes. In contrast, differentially expressed transcripts of mRNAs were highly enriched for protein metabolic process and RNA metabolic, spermatogenesis, sperm motility, cell cycle, chromatin organization, heterocycle and aromatic compound metabolic processes. Kyoto encyclopedia of genes and genomes (KEGG) pathway analysis showed that the differentially expressed lncRNAs were involved in RNA transport, mRNA surveillance pathway, PI3K-Akt signaling pathway, AMPK signaling pathway, protein processing in endoplasmic reticulum. Metabolic pathways, mRNA surveillance pathway, AMPK signaling pathway, cell cycle, RNA transport splicesome and endocytosis incorporated with the differentially expressed mRNA. Furthermore, many lncRNAs were specifically expressed in testis/sperm, and 880 lncRNAs were conserved between human and mouse. In summary, this study provides a preliminary database valuable for identifying lncRNAs critical in the late stage of spermatogenesis or important for sperm function regulation, fertilization and early embryo development.

## Introduction

Sperm are specialized cells generally considered transcriptionally and translationally inert. Early studies proposed that the lack of RNAs in sperm cells was a result of loss of most of the cytoplasm during processes in spermatogenesis. The RNAs detected in the paternal gamete were initially assumed to be either left behind after degradation and expulsion of the residual body from spermatogenesis or simply contaminants from other surrounding cells [1, 2]. However, later studies showed that sperm from mice contains about 100 fg of total RNA [3] and human sperm contains 10–20 fg of total RNA [4] in one sperm cell, which is 1% of the RNA content in somatic cells. It is well known that many RNAs, especially mRNA and small noncoding RNAs, have been investigated in mature sperm [5–7]. Nevertheless, RNAs in mature sperm are only remnants from spermatogenesis with no biological functions have aroused abroad controversy. Furthermore, recent reports have demonstrated that noncoding RNAs, such as tsRNAs and miRNAs, of sperm play important roles in paternal heredity of diet-induced obesity and metabolic disorders [8, 9], and sperm mRNAs exert considerable functions during development of early mammalian embryos [10, 11], perhaps directly controlling embryonic gene expression [12]. These studies suggested that RNAs, including noncoding RNAs, of mature sperm have important biological functions.

LncRNAs, which are commonly defined as noncoding RNAs longer than 200 bp, are novel regulatory molecules that modulate a wide variety of functions and are involved in various pathophysiologic processes and human diseases. LncRNAs are highly enriched and specifically expressed in the testes and during spermatogenesis stages [13, 14], implying that lncRNAs might play important roles in sperm production. However, the detailed profile and roles of lncRNAs in mammalian mature sperm have not been systematically investigated.

The aim of this study was to elucidate the lncRNA profile in mouse mature sperm with stranded-specific RNA-seq by Ilumina HiSeq 4000 technology. We also used bioinformatics methods and performed function predictions to screen lncRNAs candidates that are potentially involved in spermatogenesis and sperm function.

## Materials and methods

### Ethics statement

The research involving animals was conducted with the approval of the Animal Research Ethics Committee of Medical School of Nanchang University.

### Sperm collection and purification

Eight-week-old healthy male C57BL/6J mice were purchased from Hunan SJA Laboratory Animal Co., Ltd. (Changsha, China). All mice were maintained at a constant temperature (22 ± 2°C) and relative humidity (40–60%) with a 12 h light/dark schedule and free access to laboratory chow (*ad libitum*) and water. Sixty mice (10–16 weeks old) were divided into four groups to provide four pools of sperm samples from 15 mice each. The testes harvested from above-mentioned mice were arranged into two biological replicates of testes samples (n = 2). Mice were sacrificed by cervical dislocation. Sperm were collected as previously described [15] with some modifications. Briefly, the vas deferens and caudal epididymis was isolated and perforated, and then the sperm were allowed to swim out in phosphate buffered saline (PBS) a 5% CO_2_ incubator (Heal Force, Hong Kong) at 37°C for 15 min. The samples were then filtered with a 500-mesh sieve to remove the tissue debris. The sperm were then treated with somatic cell lysis buffer (0.1% SDS, 0.5% Triton X-100 in DEPC-treated H_2_O) for 30 min on ice to eliminate somatic cell contamination. Non-sperm cell contamination (< 0.2%) was checked by microscopy. After centrifugation, the top layer was carefully removed and the sperm pellet was washed twice with 10 ml of somatic cell lysis buffer at 700 g for 5 min at 4°C. Finally, the purified sperm samples were used for RNA isolation.

### RNA isolation and quality control

Total RNAs were extracted from the purified sperm (n = 4) and testes (n = 2) samples using RNeasy^®^ Plus Micro (Qiagen, Duesseldorf, Germany) according to the manufacturer’s instructions with some modifications. We added 1 ml of buffer RLT (a guanidine-thiocyanate lysis buffer supplemented with 10 mM DL-Dithiothreitol) to the sperm pellet and 10 mg testes tissues, and then homogenized the sample by passing the samples through a 20-G needle attached to a 1-ml syringe 10 to 15 times. After total RNA was bound to the membrane and other contaminants were efficiently washed away, on-column DNase digestion was performed to eliminate trace amounts DNA contamination. The concentration and integrity of the total RNA samples were evaluated using a NanoDrop ND-1000 spectrophotometer (Thermo Fisher Scientific Inc., Wilmington, DE, USA) and a 2100-Bioanalyzer with the RNA 6000 Nano Chip (Applied Biosystems, Carlsbad, CA, USA), respectively.

### Deep sequencing

Strand-specific transcriptome sequencing was conducted using Illumina HiSeq^TM^ 4000 with PE100 according to the manufacturer’s instructions developed by BGI (Wuhan, China). Briefly, the RNase H protocol was performed to remove rRNA as previously described [16]. The DNase digested RNA was purified with AMPure Beads and then fragmented to about 140–160 nt. The RNA fragments were further converted into a DNA library through end repair, adaptor ligation, reverse transcription circularization, and PCR amplification. The library was further purified with AMPure Beads and validated using the Agilent 2100 Bioanalyzer (Agilent Technologies Inc., CA, USA) and ABI StepOnePlus Real-Time PCR System (Applied Biosystems, CA, USA). The qualified library was sequenced by Illumina HiSeq^TM^ 4000.

### Bioinformatic analysis

The raw data produced by HiSeqTM 4000 was subjected to quality control tests, including removing the adaptors as well as empty reads and then filtering the low-quality reads. The clean reads were aligned to reference sequences using the SOAPaligner/SOAP2 software. The distribution and coverage of reads aligned with the reference genome were counted. The gene expression level was calculated using FPKM method [17].

FPKM = (fragments per kilobase transcriptome)/(million mapped reads)

GO analysis (http://www.geneontology.org) allows functional association of differentially expressed lncRNA-targeted mRNAs and mRNAs using three structured networks of defined terms that describe gene-product attributes. GO terms with corrected p values of less than 0.05 were considered significantly enriched by differentially expressed genes.

Kyoto Encyclopedia of Genes and Genomes (KEGG) is a database resource for understanding high-level functions and utilities of the biological system, such as the cell, organism and ecosystem, from molecular level information, especially large-scale molecular datasets generated by genome sequencing and other high-throughput experimental technologies (http://www.genome.jp/kegg/).

LncRNA-targeted genes were predicted based on the *cis* and *trans* principle. For each lncRNA, we calculated the Pearson and Spearman correlation of its expression value with that of each mRNA. The mRNAs that were co-expressed with the lncRNA of interest were defined as having Pearson correlation and Spearman correlation that exceeded 0.8 and P values less than 0.05. The mRNA loci within 10 kb upstream or 100 kb downstream of lncRNA were defined as cis-regulated target genes. RNAplex [18] was conducted on lncRNA trans-targeted genes with free energy less than -30.

### Reverse transcription (RT) and quantitative (q) PCR

The RNA was reverse transcribed to cDNA using the PrimeScript RT reagent with gDNA Eraser Kit (TaKaRa, Japan). RT-PCR was used to verify a subset of full-length transcripts (Lnc2, Lnc3, *CatSper2* and *Tssk6*) of spermatozoal RNA. PCR products were subjected to fractionation on 1.5% agarose gels at 120 V, 30 min. After separation, the gels were stained with ethidium bromide and visualized using a UV transilluminator. Images were captured digitally.

qPCR was performed with the StepOnePlus™ Real-Time PCR Systems (Applied Biosystems, CA, USA) using the SYBR Premix DimerEraser Kit (TaKaRa, Japan) according to the manufacturer’s instructions. The qPCR procedure was carried out with denaturation at 98°C for 30 s, followed by 40 cycles of denaturation at 98°C for 5 s, annealing at 60°C for 15 s, and extension at 72°C for 20 s. The expression levels of actin, *Rplp1* and *β-actin* genes were used as internal controls to normalize the related gene expression levels. A melting curve analysis was performed to check the specificity of PCR products. Three replicates were run for each sample and the qPCR assay experiment was repeated at least three times to obtain good reproducibility. The mRNA and lncRNA primer sequences used for RT-PCR and qPCR are shown in Tables 1 and 2, respectively.

**Table 1:** RT-PCR and qPCR primers of examined coding genes.

Gene symbol	NCBI no.	Tm (°C)	Length (bp)	Primers (5′→3′)
*elf3*	NM_001289613.1	62	3114	F: GGATCTTCGGACCCAGCAGAAG
				R: TGCTTTCAAATGTTTTTTCTTTATTTA
*elf3*(qPCR)	NM_001289613.1	60	128	F: CTTGGGCGTCAACGGCTACA
				R: CGCCAGCACTTCTCTCAGCAG
*atsper2*	NM_153075.3	58	2247	F: TTAGATGAACGTCCGCCGG
				R: CGCGTCAGCAGTATCTATTAGATATT
*CatSper2*(qPCR)	NM_153075.3	60	114	F: GCCTACCCAGTTTCCCATTCA
				R: CATTAGCCCAGGCAGGTTCTC
*Pcsk4*	NM_008793.2	58	2514	F: CTATATACTCTCCGCCCCCCG
				R: TTCTGTTTAACTTTTATTTGGCAAC
*Pcsk4*(qPCR)	NM_008793.2	60	114	F: AGCTTCCTGGTAACCTTTGGTC
				R: CTGTGGCTGGGTCTGACTTTG
*Tssk6*	NM_032004.2	60	1342	F: GTAATTGGGCACATGGGGGC
				R: TTTGCAAGGAAGAGCGAATTTATTG
*Tssk6*(qPCR)	NM_032004.2	62	141	F: TAAGCTGGGCCGCACGATAG
				R: CGAGGCAGGAACTTGTTGACG
*RPLP1*	NM_018853.3	60	145	F: AACATTGGGAGCCTCATCTGC
				R: CCTCGGACTCTTCCTTCTTTGC
*β-actin*	NM_007393	60	147	F: CCCATCTACGAGGGCTA
				R: GTCACGCACGATTTCC

**Table 2:** RT-PCR and qPCR primers of examined lncRNA genes.

Noncode ID	Tm(°C)	length(bp)	Primers (5′→3′)
NONMMUG056132.1(Lnc1)	60	1152	F: TACGGACAGAAGGTCTGCCC
			R: ATGGGAACTGAGAGTATATAAGAGTG
NONMMUG056132.1(Lnc1)(qPCR)	60	80	F: GACAGAAGGTCTGCCCATAGATG
			R: GGCGGAGGAGGAAGGTGAAT
NONMMUG015781.2(Lnc2)	60	410	F: ACATTTGGTGTATGTGCTTGGCT
			R: TAGATCGCAGCGAGGGAGCT
NONMMUG015781.2(Lnc2)(qPCR)	60	96	F: ACATTTGGTGTATGTGCTTGGC
			R: CCTGGGCGGGATTCTGACTT
NONMMUT020970.2(Lnc5)	58	115	F: CCTCCTTCTCATCACTTC
			R: CTCTCCATTCTTCATCCTT
NONMMUT038067.2(Lnc6)	58	153	F: GATTGCTTACTGAGAGGAT
			R: TGACACTGGCTTATATTCTAT
NONMMUT080684.1(Lnc7)	58	198	F: AGGAATGTCTGCTGAATG
			R: GAAGAGTCTTGTGAATGGA
LTCONS_00160905(Lnc8)	54	171	F: CCCCAAGATTCCTTCCTC
			R: TTCCTGCTAACCGTTCCA
NONMMUT051344.2(Lnc9)	54	196	F: GAGGGTCTGGGAGTCAAA
			R: CATTCAAGGGACAGCACA
NONMMUT071792.2(Lnc10)	54	381	F: CGGGTTCACTGGTGTAAG
			R: GCTGAATGCCTTGTCGTA
LXLOC_030768(Lnc3)	58	581	F: CGTGGAATGACCTTGTGATTCTG
			R: CTGTCCACTGCTGACTCATGTGC
LXLOC_030768(Lnc3)(qPCR)	60	143	F: CCACCCACAATAGCCACAAC
			R: CTCCGTCACCATCTTATCCAAG
LXLOC_013896(Lnc4)	62	651	F: AGCTCAGGTCCCTGCCTGG
			R: CTCCAGGTCCTCCTCAGCTCC
LXLOC_013896(Lnc4)(qPCR)	60	114	F: TCCAGCCACAGGTCAATCTACAC
			R: GAGCTCGCCTTCTGCCTAAAC

### Fluorescence in situ hybridization (FISH)

We performed lncRNA FISH staining as previously described [19, 20]. Briefly, sperm cells were fixed in methanol and acetic acid (1:3) for 20 min and permeabilized in 0.2% Triton X-100 on ice for 5 min. The cells were rinsed with 2× SSC (saline sodium citrate) prior to hybridization, and the appropriate amount of probe was applied in a hybridization solution containing 10% formamide, 2 × SSC, and 10% dextran sulfate (w/v). Hybridization was allowed to occur overnight in a humid chamber at 37°C. Cells were then washed twice for 30 min at 37°C with 10% formamide in 2 × SSC. DAPI was applied during the second wash. After washing, the coverslips containing the stained sperms were loaded on a clear slide and examined under an FV1000-IX81 confocal laser scanning biological microscope (Olympus, Japan) with an LD laser (405) for DAPI and an M_Ar laser (488 nm) for each fluorescence channel targeted with FAM-labeled lncRNA probes (BersinBio, Guangzhou, China). The probe sequences are listed here.

Lnc2: AAGACGAACGGCTCTCCGCACCGGACCCCGGTCCCGACGCCCGGCG GGGGGAGACCCCGCGCCGCCGCGGGGACGACGCGGGGACCCCGCCGAGCGGGGACGGACGGGGACCCGGCTATCCGGGGCCAACCGAGGCTCCTTCGGCGCTGCCGTATCGTTCCGCCTGGGCGGGATTCTGACTTAGAGGCGTTCAGTCATAATCCCACAGATGGTAGCTTCGCCCCATTGGCTCCTCAGCCAAGCACATACACCAAATGT

Lnc3: TTTCCCCTCGTTCTGCCAGACAACTACCTGGCTGAAGCCGTTTGGCTT GCTTCTCTGTGTCCTGCATTATTTTCTCGGCCCATTTCCAGTACCTGACACTCCTTTCATGGCTGCCAGAAGCACTTCCTCCGCCTCTGTCTTTGTAATGTAGGCTTTTGTCTTGTTCTGTCCCTCTCCTCTGTCCCAGCTCACACAGAGACAGACTCTCCGTCACCATCTTATCCAAGAGTTCATTTTTGTTGATGGTTATCCACAGTTTC

### Statistical analysis

Data are represented as mean ± standard deviation (SD). All statistical analyses were performed using SPSS 19.0 (v19.0; IBM, Chicago, IL, USA). Statistical significance was determined by the t-tests. P < 0.05 was regarded as statistically significant and P < 0.01 was considered extremely statistically significant.

### Data access

The RNA-seq data sets generated in this study have been submitted to the NCBI Gene Expression Omnibus (GEO; http://www.ncbi.nlm.nih.gov/geo) under accession numbers GSE88732.

## Results

### Purity identification and size distribution of mature sperm RNAs

**Fig 1:**
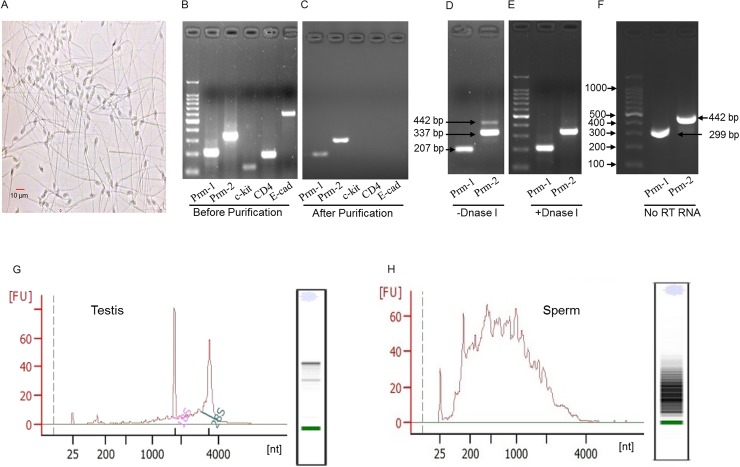
Quality control of sperm RNA. **Confirmation of sperm purity and electrophoretic size distribution of extracted total sperm RNA.** (A) Morphology of collected sperm under phase contrast microscopy. (B) Biomarkers of leukocytes (*CD4*), testicular germ cells (*c-kit*) and epithelial cells (*E-cadherin*) could be amplified easily from the RNA extracted from unpurified sperm sample. (C) After sperm was purified, non-sperm cell markers (*CD4*, *c-kit*, *E-cadherin*) were unable to be amplified from the RNA extracted from our purified sperm sample, while positive markers of sperm (*Prm1* and *Prm2*) were easily detected. (D, E) The 442 bp PCR product of *Prm2* indicated DNA contamination (D), which disappeared after DNase I digestion (E). (F) The DNA PCR product of *Prm1 and Prm2* could be amplified from no reverse transcription (RT) sperm RNA. (G, H) Electrophoretic size distribution of RNAs in mouse testis (G) and mature sperm (H) analyzed by Agilent Bioanalyzer.

The purity of mature sperm isolated from vas deferens and caudal epididymis was checked by microscopy and the results showed that non-sperm cells were less than 0.2% (Fig 1A). Biomarkers of leukocytes (*CD4*), testicular germ cells (*c-kit*) and epithelial cells (*E-cadherin*, *E-cad*) were unable to be amplified from the RNA extracted from our sperm sample, while positive markers of sperm (*protamine-1* and *-2*, *Prm1*, *Prm2*) were easily detected (Fig 1B and 1C). On the other hand, the 442 bp PCR product of *Prm2* indicated DNA contamination disappeared after DNase I digestion (Fig 1D and 1E). No reverse transcription RNA served as control to indicate DNA contamination (Fig 1F). These results confirmed the purity of our sperm sample. As shown in Fig 1G and 1H, sperm RNAs showed enrichment of smaller size fractions and lack the 28s and 18s peaks that are easily observed in testes tissue.

### Expression patterns of LncRNA and mRNA in mouse mature sperm

**Fig 2:**
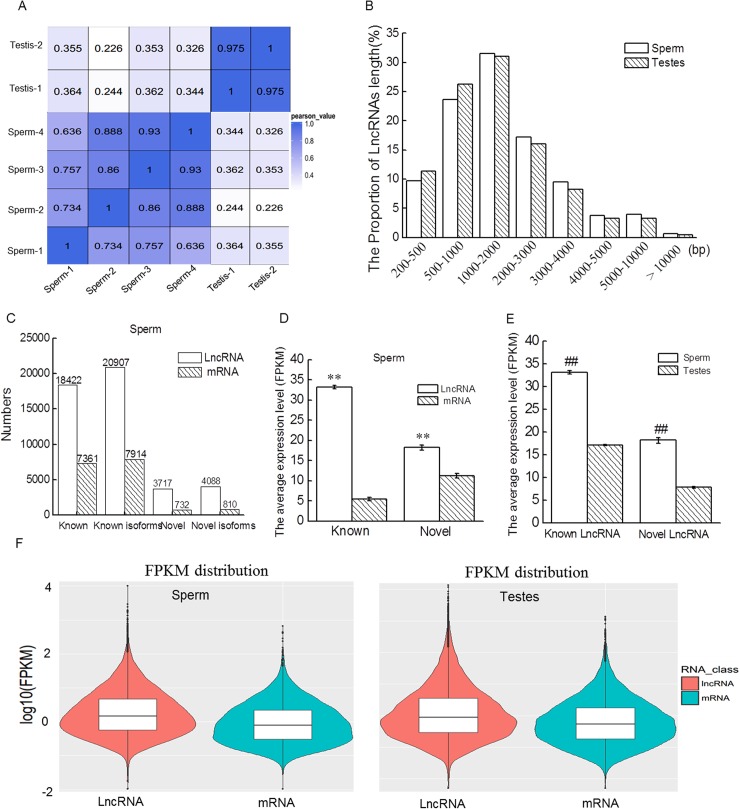
LncRNAs expression profiles in mouse mature sperm. (A) Correlation analysis of all sequencing samples. (B) The numbers of lncRNAs according to length in mice testes and mature sperm. (C) The numbers of lncRNAs and mRNAs in mature sperm. (D) The average expression levels of known and novel lncRNAs and mRNAs in mice mature sperm. (E) The average expression levels of known and novel lncRNAs in mice testes and mature sperm. (F) The violin graph of lncRNAs and mRNAs in in mice testes and mature sperm. **P < 0.01 compared with mRNA, ##*P* < 0.01 compared with testes.

To ensure the data were reliable, correlation analysis of all sequencing samples were conducted. Sperm-1, sperm-2, sperm-3 and sperm-4 indicate four biological replicates for sperm, and testis-1 and testis-2 were two biological replicates of testes. As shown in Fig 2A, the data of sperm-1 was aborted as the Pearson coefficient < 0.85 compared with other biological replicates of sperm groups. After assembly and analysis, we found that the length of most lncRNAs ranged from 200 bp to 3,000 bp (82.08%) in sperm, with a similar distribution observed in testes (Fig 2B). We identified 20,907 known lncRNA transcripts from 18,422 known annotated lncRNA genes and predicted 4,088 novel lncRNA transcripts from 3,717 lncRNAs with the Coding Potential Calculator (Fig 2C) in mature sperm in mice. The numbers and average expression level of lncRNAs were higher than those of mRNAs in mature sperm (Fig 2D and 2F). Furthermore, compared to testes, the average expression level of lncRNAs was also higher in sperm (Fig 2E).

### Integrity evaluation and existing forms identification of sperm LncRNAs

**Fig 3:**
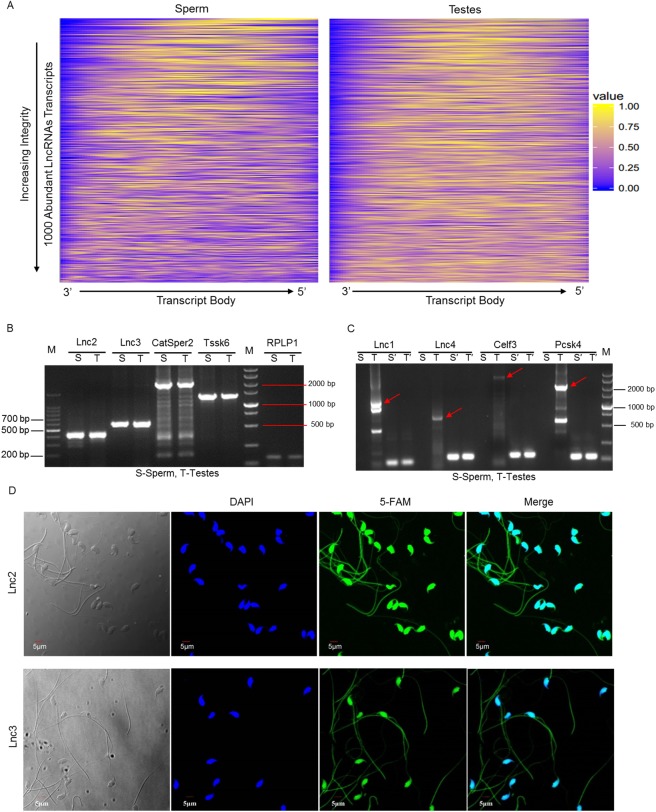
Integrity evaluation and existing forms of sperm lncRNAs. (A) Integrity evaluation of sperm lncRNAs using a computational approach. To determine the uniformity of coverage for each, RNA transcripts were divided into 100 bins, and a 5-bin moving average was used to calculate localized variations in sequencing coverage. The squared deviation from expected coverage for each bin was summed and used as an intactness score to rank the 1000 lncRNAs according to their stability. (B) Intact RNAs are present in sperm; the testes served as positive control to amplify the proposed full-length mRNAs and lncRNAs. (C) The fragmented RNAs degraded or cleaved from the intact transcripts were amplified in sperm. S′ and T′: Representative fragments were amplified from sperm and testes cDNA, respectively. (D) The localization of two representatively intact lncRNAs with FISH staining.

A computational approach was developed to globally identify the population of intact RNAs in mature sperm. This analysis was predicated on the assumption that sequencing coverage would vary less across the length of an intact transcript relative to one that was fragmented [2]. The 1000 most abundant lncRNAs from testes-1 and sperm-2 samples were used to establish a means of measuring individual transcript integrity (Fig 3A, S1 Table and S2 Table). As shown in Fig 3B, sperm-specific RT-PCR was used to verify that spermatozoal RNA expressed full-length transcripts (such as Lnc2, Lnc3, *CatSper2* and *Tssk6*). RNAs that are not intact transcripts might be degraded or cleaved to small fragments (Fig 3C). Furthermore, FISH staining results confirmed that two representatively intact lncRNAs (Lnc2 and Lnc3) were expressed in sperm (Fig 3D).

### Analysis of tissue-specific and conserved lncRNA in sperm

**Fig 4:**
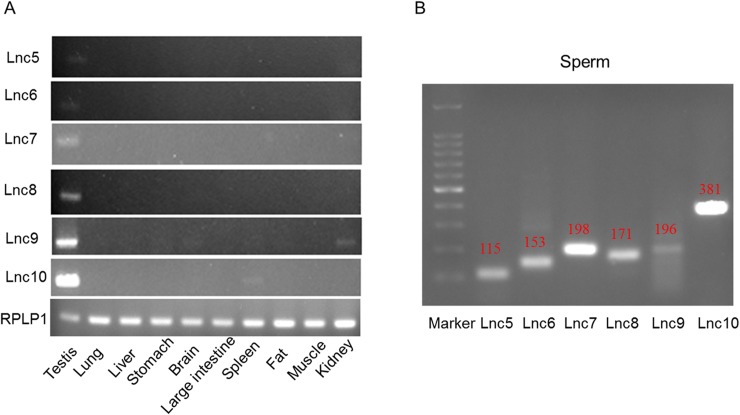
Validation of tissue-specific expression of lncRNA by RT-PCR. Total RNA of testis, lung, liver, stomach, brain, large intestine, spleen, fat, muscle, kidney, and sperm were isolated using RNeasy® Plus Micro kit, and RT-PCR was performed to detect the lncRNA candidates expression level. Rplp1 genes were used as loading controls.

We compared lncRNA profiles of sperm and testes with other 6 tissues (heart, hippocampus, liver, lung, spleen, and thymus) lncRNA profiles abstained from noncode database, and screened the testes and/or sperm-specific expression lncRNA based on the FPKM. The results showed that 6,983 lncRNA (3,575 annotated lncRNA and 3,408 predicated novel lncRNA) exclusive expressed in testis and sperm (S3 Table), 6 of which were confirmed by RT-PCR (Fig 4). LiftOver tool from UCSC was used to analyze the conservative of lncRNA between human (Data not shown) and mouse. 880 lncRNAs were conserved between human and mouse with > 80% overlap (S4 Table).

### Functional LncRNA prediction in mature sperm

**Fig 5:**
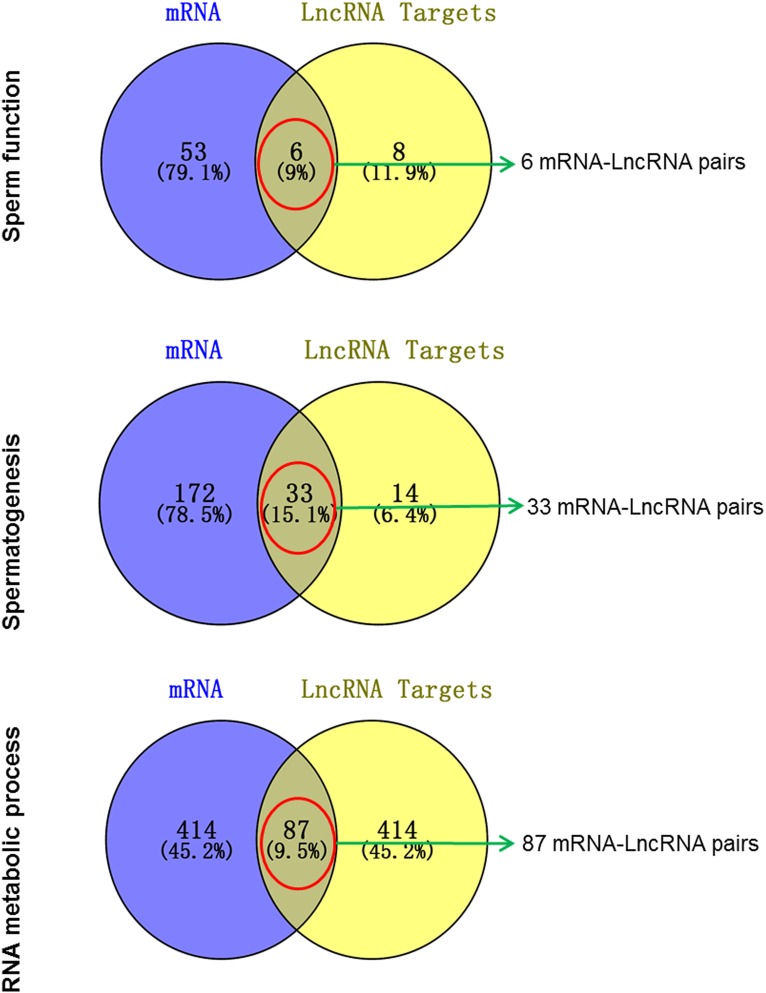
The putative functional lncRNAs in mature sperm. LncRNA target genes were predicted based on the “Cis and Trans” RNA-RNA interaction principle. One-to-one pairs of lncRNA and mRNA were deemed to putative functional lncRNAs modulating the spermatogenesis or sperm function.

We performed GO and KEGG enrichment analysis of the differentially expressed genes between sperm and round spermatid [21]. The differentially expressed coding genes that were also differentially expressed lncRNA targets were screened as putative candidates of functional lncRNAs in the late stage of spermatogenesis or in mature sperm. Three GO terms, including sperm function, spermatogenesis and RNA metabolic process, were selected to predict the functional lncRNAs in the late stage of spermatogenesis or in mature sperm (Fig 5). We identified 6, 33 and 87 overlapped coding genes, respectively, which corresponded to lncRNAs that might be candidates that modulate sperm mature or RNA metabolic process via targeted coding genes. S5 Table lists 120 of the lncRNA and mRNA pairs.

### Enrichment analysis of differentially expressed genes between sperm and round spermatid

We compared the lncRNA and mRNA expression levels in the sperm of our data and round spermatid groups from GEO database (GSM1968532 and GSM1968533). We identified 4,040 upregulated lncRNAs and 4,261 downregulated lncRNAs (S6 Table) and 1,280 upregulated mRNAs and 3,619 downregulated mRNAs (S7 Table) in the sperm group compared with the round spermatid group with a set filter of fold-change > 2.0 (*P* < 0.05).

To characterize the function of lncRNAs in the late stage of spermatogenesis or in mature sperm, we attempted to predict the cis- and trans-regulated target genes of differentially expressed lncRNAs. Based on the “Cis and Trans” principle, we identified 14,259 targeted genes of lncRNAs. In terms of GO terms, differentially expressed transcripts of mRNAs genes were highly enriched for protein metabolic process and RNA metabolic, spermatogenesis, sperm motility, cell cycle, chromatin organization, heterocycle and aromatic compound metabolic processes et al. For the differentially expressed lncRNA targeted genes, highly enriched GO terms included nucleic acid metabolic, protein modification, chromatin and histone modification, heterocycle compound metabolic, sperm function, and other processes.

Based on our KEGG pathway analysis, the most enriched pathways for the differentially expressed mRNAs were mainly related to the metabolic pathways, mRNA surveillance pathway, cell cycle, RNA transport and endocytosis et al. For the differentially expressed lncRNA targets, the most significant pathways were those involved in RNA transport, PI3K-Akt signaling pathway, AMPK signaling pathway, protein processing in endoplasmic reticulum, and some other processes.

### qPCR validation

**Fig 6:**
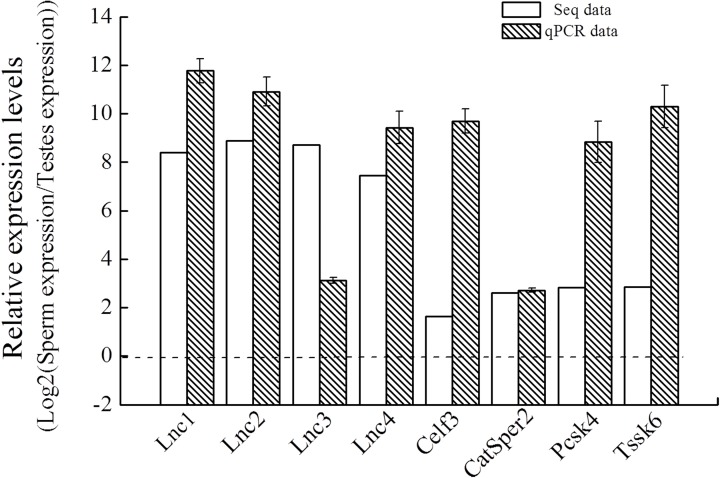
qPCR validation of the upregulated RNAs. After total sperm RNA was isolated, qPCR was performed to detect the RNA expression in sperm and testes. *Rplp1* and *β-actin* genes were used as loading controls to normalize RNA expression levels. Data are expressed as the mean ± standard deviation (n = 3).

To confirm the reliability of our sequencing data, we randomly selected four upregulated lncRNAs and mRNAs from the pool of mRNAs and lncRNAs whose fold changes were > 2.0, and analyzed their expression levels by qPCR in testes and sperm samples. As shown in Fig 6, the qPCR results were consistent with the sequencing data and showed the same trend of upregulation for each RNA.

## Discussion

The importance and potential function of lncRNAs in male sterility have been the subject of attention, as lncRNA expression levels are highly enriched and specific in testes and during spermatogenesis stages [10, 14], and a large number of lncRNAs have been identified from the reproductive system including testes tissue [22] and germ cells of Drosophila [23], mouse [24], rat [25] and human [26]. One recent report examined the lncRNA expression in mice sperm [27], but the results were unreliable because of somatic cell contamination. Furthermore, the authors only focused on known lncRNAs using microarray analysis. To the best of our knowledge, this was the first study to comprehensively identify and characterize lncRNAs in mouse sperm.

Sperm obtained from epididymis and vas deferens often contain some somatic cells like epithelial cells, testicular germ cells and leukocytes. Since sperm RNA content is about 1% of that in somatic cells and DNA quantity in sperm is very high with respect to RNA, the precaution of contamination caused by somatic cell RNAs and sperm DNA is very critical. In our study, we obtained high purity sperm RNA as indicated by the inability of PCR amplification of biomarkers of leukocytes (*CD4*), testicular germ cells (*c-kit*), epithelial cells (*E-cadherin*, *E-cad*) and genomic DNA products of *Prm2*. In addition, RNA-seq in sperm is far more complex than that in somatic cells because the intrinsic characteristics of spermatozoal RNA were mainly fragmented [28] and displayed an unusual 28S/18S rRNA ratio [29]. These results are consistent with our findings that sperm RNAs appeared as a complex mixture of fragmented products and some intact RNAs that ranged about from 25 to 4,000 nt.

Sperm RNAs possess abundant alternative isoforms, resulting in higher numbers of transcripts than genes [2]. In the present study, we identified 20,907 known lncRNA transcripts from 18,422 known annotated lncRNA genes and predicted 4,088 novel lncRNA transcripts from 3,717 lncRNAs in mature mice sperm. Many lncRNA genes could transcribe two or three transcripts (data not shown), which might result in quantitative modulation of gene expression in spermatogenesis through cotranscriptional coupling mechanisms [30, 31]. Furthermore, the number of lncRNAs alternative splicing events was higher than that of mRNA. Surprisingly, although the numbers of lncRNAs were higher than mRNAs, the average expression level of lncRNAs was higher than mRNAs in mature sperm. This is different from the other tissues, in which the average expression level of lncRNAs is lower than mRNAs. Furthermore, compared to testes, the average expression level of lncRNAs was higher in sperm, which furthers the idea that lncRNAs might have potential functions in mature sperm.

According to the assembly and alignment results, the length of most lncRNAs ranged from 200 bp to 3,000 bp (82.08%) in sperm, with a distribution similar to that of testes. However, as mentioned above, many sperm RNAs are fragmented, and therefore we developed a computational approach to globally evaluate the integrity of lncRNAs. As expected, the integrity ratio of sperm lncRNAs was lower than that of testes. Furthermore, two mRNAs and two lncRNAs of interest were selected from the top 100 most intact profiles to test *in silico* predictions of stability by RT-PCR. The full length mRNA of *CatSper2* and *Tssk6* were detected in sperm. Two lncRNAs, Lnc2 and Lnc3, were also present in intact forms. FISH results further confirmed that Lnc2 and Lnc3 were present in sperm. However, some transcripts, such as the mRNAs of *Celf3* and *Pcsk4* and lncRNAs of Lnc1 and Lnc4, were fragmented because we had not amplified the full-length production of these transcripts but only identified the partial production that might be derived from fragmented RNAs in sperm. Similarly, three intact mRNAs, *NDUFA13*, *IZUMO4* and *CIB1*, were identified in sperm by Sendler, et al. Since the stability and integrity of RNA were functionally correlated [2], the existence of intact lncRNAs in sperm indicates that lncRNAs might exhibit regulatory effects on sperm function, a topic worthy for deeper investigation.

Testis-specific lncRNA might be important for male infertility [22]. In the present study, many testis/sperm-specific lncRNAs were also identified, and 6 of which were confirmed by RT-PCR. Furthermore, although conservative of lncRNA was lower than mRNA, 880 high conserved lncRNAs between human and mouse were found. These testis/sperm-specific or conserved lncRNAs should be concerned and investigated to elucidate the function of lncRNAs in mature sperm.

Sperm might retain certain transcriptional and translational activities [32, 33], which might result in the intact RNAs function in regulating targeted gene expression in sperm. The knockdown of coding genes retained in sperm could impair the sperm function [34, 35]. Hence, the putative functional lncRNAs that affect nucleic acid metabolic processes might affect the spermatogenesis or mature sperm function via modulating corresponding target genes. The nucleic acid metabolic process genes, including RNA metabolic process and ncRNA processing related genes, might be important for sperm mature and sperm function because cleavage of rRNA ensures translational cessation [36] and maintenance of intact RNAs safeguarded sperm function and embryonic development when sperm are delivered at fertilization [10]. KEGG analysis also confirmed that mRNA surveillance pathway-related mRNA and lncRNA genes are important for sperm function. These might explain why well-controlled degradation and retention of RNAs are important for development of fertile sperm. Collectively, the lncRNAs that regulate sperm function, spermatogenesis and RNA metabolic process related coding genes and lncRNAs genes should be examined in future studies for uncovering the roles of lncRNAs in the late stage of spermatogenesis (elongation and maturation of sperm) or in mature sperm.

In our study, eight differently expressed genes that were identified by sequencing data were further corroborated by qPCR results, which suggested that sequencing data were reliable. It is likely that the dis-regulated RNAs in sperm, compared to round spermatid, are required earlier in spermatogenesis, and that RNAs associated with nuclear organization and the chromosome are no longer required in fully differentiated sperm and are simply residual. However, some abundant sperm transcripts may also function in the oocyte upon fertilization [37, 38] and zygotic successful development [10]. Therefore, we proposed, on one hand, that the content of RNAs might reflect the disorder and errors in the late stage of spermatogenesis, including sperm maturation, resulting from abnormal RNA processing and metabolism, and further explain the infertility mechanisms. On the other hand, some RNA elements (fragments and intact transcripts) present in sperm could be used as markers to assess the fertility status and outcome of assisted reproductive technologies [39]. Collectively, these differently expressed RNAs might show some function in sperm maturation, within the sperm or in future post-fertilization events.

## Conclusions

In summary, the present study systematically identified and characterized many lncRNAs in mouse mature sperm. These lncRNAs were detected in sperm as fragmented and intact forms. The abundant, intact and testis/sperm specific lncRNAs related RNA metabolism, spermatogenesis, and sperm function represent good candidates to elucidate the functions of lncRNAs in mature sperm. Our results will contribute to the current understanding of the lncRNA complexity in mature sperm and provide a preliminary database of sperm lncRNAs for investigating their regulatory roles in sperm.

## Supporting information

S1 Table Integrity evaluation of sperm lncRNAs. (XLS)

S2 Table Integrity evaluation of testis lncRNAs. (XLS)

S3 Table Tissue-specific lncRNA in testis and sperm. (XLS)

S4 Table The conserved lncRNA between human and mouse. (XLS)

S5 Table 120 of the lncRNA and mRNA pairs. (XLS)

S6 Table The differential expression of lncRNA genes between sperm and round spermatid. (XLS)

S7 Table The differential expression of mRNA genes between sperm and round spermatid. (XLS)
